# The Role of Quantitative PCR in Evaluating the Clinical Significance of Human Bocavirus Detection in Children

**DOI:** 10.3390/v16101637

**Published:** 2024-10-19

**Authors:** Maja Mijač, Tomislav Meštrović, Irena Ivković-Jureković, Tatjana Tot, Jasmina Vraneš, Sunčanica Ljubin-Sternak

**Affiliations:** 1 Clinical Microbiology Service, Dr. Andrija Štampar Teaching Institute of Public Health, 10000 Zagreb, Croatia; maja.mijac@stampar.hr (M.M.); jasmina.vranes@stampar.hr (J.V.); sljsternak@stampar.hr (S.L.-S.); 2 Medical Microbiology Department, School of Medicine, University of Zagreb, 10000 Zagreb, Croatia; 3 University Centre Varaždin, University North, 42000 Varaždin, Croatia; 4 Institute for Health Metrics and Evaluation, University of Washington, Seattle, WA 98195, USA; 5 Department for Health Metrics Sciences, School of Medicine, University of Washington, Seattle, WA 98195, USA; 6 Department of Pulmonology, Allergy, Immunology and Rheumatology, Children’s Hospital Zagreb, 10000 Zagreb, Croatia; irena.ivkovicjurekovic@kdb.hr; 7 Faculty for Dental Medicine and Healthcare, School of Medicine, Josip Juraj Strossmayer University of Osijek, 31000 Osijek, Croatia; 8 Department of Microbiology, General Hospital Karlovac, 47000 Karlovac, Croatia; tatjanatotka@gmail.com

**Keywords:** human bocavirus, viral load, acute respiratory infection, lower respiratory tract infection, pediatric infections, polymerase chain reaction (PCR), molecular diagnostics, co-detection

## Abstract

Human bocavirus (HBoV) has emerged as a significant pathogen primarily associated with respiratory infections in children. This study aimed to evaluate the clinical relevance of HBoV infection by quantifying viral loads in nasopharyngeal swabs from hospitalized children with acute respiratory infections (ARIs) and investigating correlations with clinical outcomes. A total of 957 children were tested, with HBoV detected in 73 cases (7.6%), either as a sole infection or co-infection with other respiratory viruses. Quantitative polymerase chain reaction (qPCR) was employed to measure viral load, and a threshold of 10^4^ copies/mL was used to differentiate high and low viral concentrations. Results have shown that children with lower respiratory tract infections (LRTIs) had significantly higher viral loads, most notably in cases where HBoV was the sole pathogen. Additionally, children with pre-existing health conditions were more likely to have elevated HBoV concentrations compared to those who were previously healthy. Children with higher viral loads were more likely to require oxygen supplementation and receive empirical antibiotic therapy, indicating a more severe clinical course. This study underscores the importance of considering HBoV viral load in clinical diagnostics, as higher concentrations were associated with more severe presentations, including the need for oxygen support. Future research should focus on refining diagnostic thresholds and exploring HBoV’s role in co-infections to enhance patient care strategies.

## 1. Introduction

Human bocavirus (HBoV) was identified nearly two decades ago [1,2,3], with manifold studies validating its capacity to cause illness primarily in children [4]. HBoV is classified into the *Parvoviridae* family and Bocaparvovirus genus, with two species that infect humans: Primate bocaparvovirus 1 that contains HBoV 1 and HBoV 3, and Primate bocaparvovirus 2 containing HBoV 2 and HBoV 4. Epidemiological research identified HBoV 1 as a prevalent cause of respiratory infections, while HBoVs 2–4 were found in the stools of individuals with diarrhea or asymptomatic subjects. Among hospitalized children, the most frequent diagnoses associated with HBoV 1 include rhinitis, acute otitis media, pneumonia, bronchiolitis, as well as acute asthma exacerbations [5,6,7,8,9,10]. Earlier studies approximated the worldwide occurrence of HBoV 1 to be around 6% [11], whereas a recent meta-analysis conducted by Polo et al. revealed an even higher proportion of positive samples among children presenting with respiratory tract infections in hospitals, almost 10% in the European population [12]. However, diagnosing this virus in clinical settings remains challenging and often inaccessible.

Laboratory diagnosis of HBoV infection relies on methods for direct virus detection [3,13,14]. Since HBoV cultivation is cumbersome, practical diagnostics primarily involve the detection of HBoV through qualitative polymerase chain reaction (PCR) or *real-time* PCR. While the virus is included in certain diagnostic panels for respiratory infections, simply detecting viral genetic material through qualitative methods is not enough to determine its clinical relevance. Namely, viral deoxyribonucleic acid (DNA) can be excreted for an extended period after infection—up to 75 days in nasopharyngeal samples of immunocompetent individuals [15]—and can also be found in asymptomatic individuals [16], indicating that PCR detection alone does not necessarily confirm the virus as the cause of the disease [17].

Moreover, interpreting positive PCR results for HBoV is hampered by the frequent co-detection of this virus alongside other respiratory viruses, making it difficult to determine its clinical significance and reflecting the complexity of diagnosing HBoV-related illnesses. Co-detection rates vary widely, ranging from 8.3% to 100%, with mean values of 52.4%, according to Guido et al. [11], or more than 80%, according to Jaartti et al. [2].

Therefore, a different diagnostic approach should be considered. For instance, quantitative PCR (qPCR) has been proposed as a more appropriate method, compared to conventional PCR, with a clinically significant viral load defined as 10^4^ or 10^6^ copies of viral DNA per mL of sample [4,18].

To better understand the clinical relevance of viral quantification, this study quantified HBoV in nasopharyngeal swabs collected from hospitalized children with acute respiratory infections (ARIs) and compared these findings with clinical factors—including infection localization, the need for oxygen support, the use of empirical antibiotic treatments, as well as the length of hospital stay.

## 2. Materials and Methods

### 2.1. Patients and Samples

This study included children hospitalized with ARI at the Children’s Hospital Zagreb (KDB Zagreb) and General Hospital Karlovac (OB Karlovac) from May 2017 to March 2021. The inclusion criteria were as follows: patients under 18 years old, ARI of suspected viral origin, and symptoms present for no more than five days prior to admission. For each child meeting the inclusion criteria, a sterile flocked swab set designed for PCR testing was used to collect combined nasopharyngeal and throat samples. These samples were placed in a viral transport medium (UTM^®^, Copan, Brescia, Italy) on the day of admission. The collected samples were stored at +4 °C until transport and promptly delivered to the laboratory (within 24 h), where they were stored at –80 °C until further processing.

### 2.2. HBoV Detection by Multiplex PCR

Extraction of viral nucleic acids was performed using the Ribospin™ vRD kit (Gene All Biotechnology, Seoul, Republic of Korea) from 300 μL of transport medium according to the manufacturer’s instructions.

The PCR for detecting nucleic acids of 15 respiratory viruses was performed using the multiplex RT-PCR method with the Seeplex^®^ RV15 OneStep ACE Detection kit (Seegene, Seoul, Republic of Korea) on the Thermal Cycler GeneAmp^®^ 9700 PCR System (Applied Biosystems, Foster City, CA, USA). Multiplex PCR and complementary DNA (cDNA) synthesis were conducted in three separate tubes with three sets of primers (A, B, and C). Each set contained primers for five viruses, and HBoV primers specific to the genus were included in set B [19]. The Seeplex^®^ RV15 OneStep ACE Detection kit included two internal controls: PCR control and WPC, ensuring the integrity of the entire process. The detection limit was 100 copies/reaction.

The amplification products were detected using the microchip electrophoresis method on the MCE^®^-202 MultiNA device (Shimadzu, Kyoto, Japan), which included software analysis in the form of electropherograms and a virtual gel.

### 2.3. HBoV Quantification

For samples where HBoV was detected using multiplex PCR, quantitative PCR was performed on the Lightcycler^®^ 480 Real-time PCR Instrument (Roche Diagnostics, Rotkreuz, Switzerland) using the LightMix^®^ Modular Bocavirus kit (Roche, TIB Molbiol, Mannheim, Germany), determining the amount of viral DNA in the sample (i.e., viral load).

The PCR primers BoF and BoR, as well as the hydrolysis probe BoP, specific to the NP-1 gene of HBoV, were used in the test, as shown in Table 1. This assay detects 10 genome equivalent copies or less per reaction (DNA dilution).

### 2.4. Clinical Data

Clinical data were obtained from patient charts and included information on comorbidities, discharge diagnoses, and inflammation markers such as C-reactive protein (CRP) and white blood cell count at the time of admission. Additional details were gathered on routine microbiological and radiological tests conducted during the hospital stay, as well as the treatment regimen, which encompassed the need for oxygen supplementation, antibiotic therapy, bronchodilator use, and corticosteroid administration. Data also covered whether patients were admitted to the intensive care unit, required mechanical ventilation, and the overall length of hospitalization.

### 2.5. Statistical Analysis

To differentiate between high and low viral loads, a cut-off value of 10^4^ copies/mL was used. To assess statistically significant associations between two categorical variables, Pearson’s χ^2^ test and Fisher’s exact test were employed based on sample size. The Mann–Whitney test was used for analyzing independent groups of data that were not normally distributed. In addition, Spearman’s rank correlation test was utilized to assess the relationship between two variables when the data did not meet the assumptions of normality or linearity, providing a robust method for analyzing monotonic relationships between variables. All analyses were conducted using the freely available statistical software R (version 4.2.3, R Core Team, http://www.r-project.org, accessed on 30 September 2024). A *p*-value of less than 0.05 (two-tailed) was considered statistically significant.

### 2.6. Ethical Approval

This study was part of the HRZZ RESPIVIRUS IP-2016-06-7556 research project titled “New and neglected respiratory viruses in vulnerable group of patients”. Conducted in accordance with the Helsinki Declaration guidelines, this study received approval from the Ethics Committee of the Teaching Institute of Public Health “Dr. Andrija Štampar” (protocol number 500-04/15-01/01; approval date 23 May 2016). Informed consent was obtained from the parents/guardians of each child, and all necessary ethical approvals were secured from the participating institutions. This ensured that participants’ rights and welfare were prioritized throughout this study, in accordance with both local and international ethical standards.

## 3. Results

**Figure 1:**
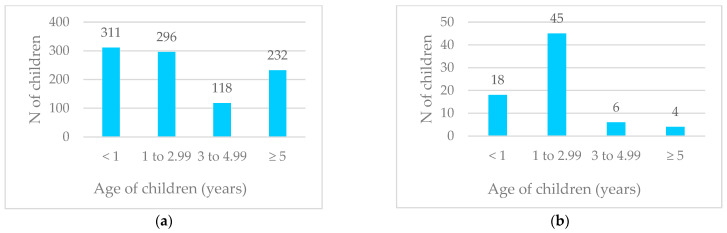
(**a**) Age distribution of all tested children (n = 957); (**b**) age distribution of children positive for HBoV (n = 73).

Over the course of four years, nasopharyngeal and throat swabs were collected from 957 children. The median age of the children tested was 1.86 years, with the youngest being just one day old and the oldest being 17 years old (Figure 1a).

Of these, 73 children (73/957) tested positive for HBoV, comprising 32 girls and 41 boys and yielding a frequency rate of 7.6% (95% confidence interval [CI]: 0.0594–0.093). In the majority of cases (60/73, 82.2%), HBoV was detected alongside at least one other respiratory virus, while in 13 samples (17.8%), HBoV was the sole pathogen identified.

The median age of children who tested positive for HBoV was 1.4 years, with an interquartile range (IQR) from 1.017 to 2.127 years, spanning from 7 days old to 15 years old (Figure 1b). The majority of children positive for HBoV were between one and three years old (45/73, 62%), with a prevalence of 15.2% (45/296) in this age group. In the three-to-five-year-old group, the prevalence was 5.1% (6/118), while in children under the age of one, it was 5.8% (18/311). Only four children with confirmed HBoV were older than five years, resulting in a prevalence of 1.2% (4/232) in this age group. The prevalence of HBoV varied significantly across different age groups (*p* < 0.001).

### 3.1. Clinical Course of Children Positive for HBoV

Out of the 73 children who tested positive for HBoV, 30 (41.1%) exhibited symptoms of upper respiratory tract infections (URTIs), while 43 children (58.9%) presented with lower respiratory tract infections (LRTIs). The clinical presentations of these cases varied widely, reflecting the different localizations and severity of the infections. The children with URTI primarily experienced conditions such as pharyngitis, tonsillitis, and rhinitis, while those with LRTI displayed more severe illnesses like bronchitis, bronchiolitis, and pneumonia. Detailed information on each child’s clinical diagnosis, the specific location of the infection (upper vs. lower respiratory tract), the most prominent symptoms at the time of hospital admission, and the findings from physical examinations are summarized in Table 2.

Among the 73 children who tested positive for HBoV, 13 (17.8%) of them had underlying comorbidities, further complicating their clinical presentation. These comorbidities included a range of chronic and congenital conditions that potentially increased their vulnerability to respiratory infections. Specifically, seven children presented with malformations of the digestive and cardiovascular systems, one child was diagnosed with cystic fibrosis, one had cutaneous mastocytosis, one had Down syndrome, one was born prematurely at 29 weeks, and two of them had documented recurrent bronchoconstriction.

**Figure 2:**
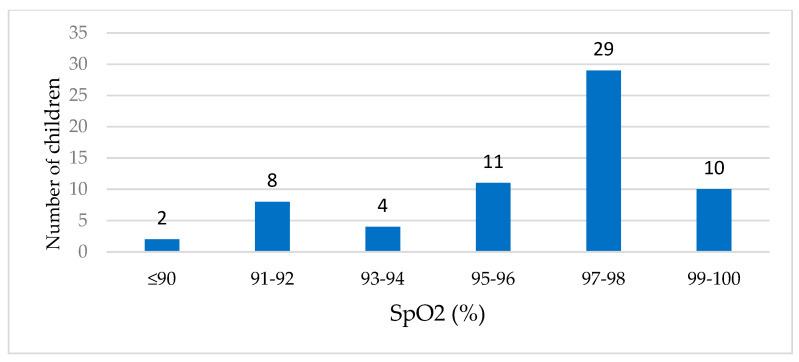
Measured oxygen saturation (SpO_2_) values in children positive for HBoV (n = 64).

Oxygen saturation (SpO_2_) in peripheral arterial blood was measured in 64 children (64/73, 87.8%) infected with HBoV. Most children had normal or slightly reduced oxygen levels; however, the lowest recorded saturation was 75% in a child who required admission to the intensive care unit for respiratory support. Figure 2 illustrates the distribution of SpO_2_ levels, highlighting the severity of oxygen desaturation in certain cases.

**Figure 3:**
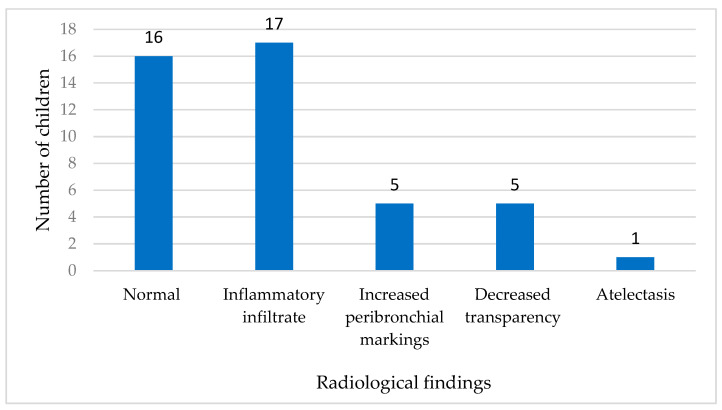
Findings of radiological examination in children positive for HBoV (n = 44).

Radiological examination (either chest X-ray or lung ultrasound) was deemed necessary for 44 out of 73 (60.3%) children with a positive HBoV finding to evaluate the extent of respiratory involvement. Among the children who underwent radiological assessment, 16 (36.4%) showed normal results, indicating no visible signs of lung pathology; however, 28 children (38.4%) exhibited radiologically confirmed pathological changes. These changes included inflammatory infiltrate in 17 children, increased peribronchial markings in five cases, and decreased transparency in five cases, while one child showed evidence of atelectatic changes in the lungs (Figure 3).

Microbiological testing was routinely performed during the hospital stay for 45 children, representing 61.6% of the total study population. Among them, one child had *Streptococcus pneumoniae* confirmed in blood culture, and another child showed coagulase-negative staphylococcus (CoNS) in blood culture. *Streptococcus pyogenes* was isolated from the throat swab of one child, while two children had *Haemophilus influenzae* and *Moraxella catarrhalis* isolated from nasopharyngeal swabs. Additionally, *Escherichia coli* in significant numbers (i.e., more than 10^5^ CFU/mL) was found in the urine sample of another child.

Regarding inflammatory markers, the median CRP value in children positive for HBoV was 15.5 mg/L, while the median leukocyte count was 13.6 × 10^9^/L.

Table 3 outlines the various therapeutic interventions administered during hospitalization for children who tested positive for HBoV. Among these, one child required intensive care for a period of four days due to severe respiratory distress. In this particular case, routine microbiological testing using an antigen-based assay from a nasopharyngeal swab initially indicated the presence of influenza A virus. However, further analysis using multiplex PCR revealed that HBoV was the sole pathogen detected, with no evidence of influenza A. Quantitative PCR confirmed a high viral load of HBoV in this child, measuring 4.4 × 10^6^ copies/mL. Despite the need for intensive care, none of the children with confirmed HBoV, including this case, required mechanical ventilation during their hospital stay.

### 3.2. Quantitative PCR Results for Children with HBoV and Localization of Infection

Quantitative PCR was performed on 73 samples that tested positive for HBoV, resulting in successful amplification in 66 samples (90.4%). The viral load in these samples ranged from 9.7 to 2.1 × 10^7^ copies/mL, with a median of 3.9 × 10^4^ copies/mL. Among these, 39 samples (53.4%) exhibited high viral loads, defined as exceeding 10^4^ copies/mL, while 34 samples (46.6%) showed lower concentrations, falling below the 10^4^ copies/mL threshold (Supplement Table S1). In samples where HBoV was the sole virus detected (mono-detection), the median concentration was 2.9 × 10^5^ copies/mL.

Out of the 73 samples tested, seven (9.6%) initially positive for HBoV in the multiplex PCR test showed negative results in the quantitative PCR test. In all seven instances, HBoV was initially detected alongside another virus when the multiplex PCR test was conducted (co-detection).

The median concentration of HBoV for patients with upper respiratory tract infection (URTI) was 8.2 × 10^3^ copies/mL (lower bound of IQR 2.8 × 10^2^, upper bound of IQR 1.1 × 10^6^). The median concentration of HBoV in cases of lower respiratory tract infection (LRTI) was 5.2 × 10^4^ copies/mL (lower bound of IQR 5.8 × 10^2^, upper bound of IQR 1.6 × 10^6^). However, when all HBoV-positive cases—both mono-detection and co-detection—were considered together, no statistically significant difference in viral load between URTI and LRTI cases was observed (*p* = 0.322).

In contrast, when analyzing only the mono-detection cases, a clearer distinction emerged. For children with URTI in whom only HBoV was detected, the median viral load was 2.5 × 10^3^ copies/mL (lower limit of IQR 3.8 × 10^2^, upper limit of IQR 4.7 × 10^3^), while those with LRTI had a median viral load of 2.3 × 10^6^ copies/mL (lower limit of IQR 7.5 × 10^5^, upper limit of IQR 6.2 × 10^6^). This difference, with a *p*-value of 0.016, suggests that significant HBoV detection, namely high viral load in nasopharyngeal secretions (NPSs), is associated with LRTI but not URTI.

**Figure 4:**
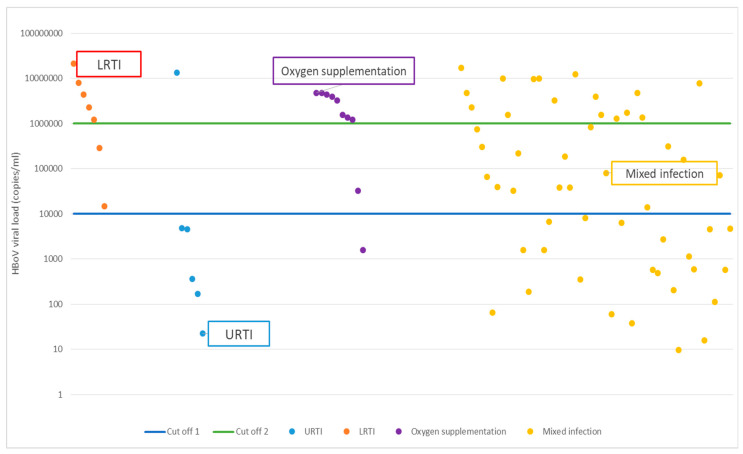
Concentration of HBoV in nasopharyngeal secretions of children with upper respiratory tract infection (URTI) and no other virus detected, with lower respiratory tract infection (LRTI) and no other virus detected, those who needed oxygen supplementation, and those with mixed infections (HBoV qPCR: Result of HBoV concentration; Cut-off 1 = 10^4^ copies/mL, Cut-off 2 = 10^6^ copies/mL).

These findings are visually represented in Figure 4, which graphically illustrates the viral concentration thresholds. The first threshold, set at 10^4^ copies/mL, was used in this study’s analysis to distinguish between high and low viral loads. The second threshold, set at 10^6^ copies/mL, is derived from the existing literature and indicates an even more clinically significant viral burden.

### 3.3. Quantitative PCR Results for Children with HBoV and Respiratory Insufficiency

Among the 73 children with confirmed HBoV, 10 children (13.7%) developed respiratory insufficiency that required oxygen supplementation during treatment. Quantitative PCR showed that in 9 out of these 10 cases, HBoV was present in high concentrations (exceeding 10^4^ copies/mL), with concentrations surpassing 10^6^ copies/mL in 8 samples (Figure 4).

### 3.4. Quantitative PCR Results for Children with HBoV Who Received Empirical Antibiotic Therapy

As previously noted, antibiotics were prescribed to 36 out of the 73 children (49.3%) who tested positive for HBoV. A statistically significant difference in viral concentration was observed between children who received antibiotic treatment and those who did not. This difference was evident in both cases where HBoV was the sole pathogen (*p* = 0.015) and in cases where HBoV was co-detected with another virus (*p* = 0.049).

### 3.5. Quantitative PCR Results for Children with HBoV and Comorbidities

Among the 13 children with comorbidities, 10 (76.9%) exhibited high concentrations of HBoV in their samples, with a median concentration of 7.57 × 10^5^ copies/mL (lower bound of IQR 7.24 × 10^4^, upper bound of IQR 1.37 × 10^6^). In contrast, children without comorbidities had significantly lower viral loads compared to those with pre-existing health issues (*p* = 0.017).

### 3.6. Quantitative PCR Results for Children with HBoV and Length of Stay in Hospital

The overall mean length of hospital stays for all children included in this study was 7.5 days. However, children with confirmed HBoV infection had shorter average hospital stays of 5.46 days. Among these, those with HBoV as the sole pathogen (mono-detection) had an average stay of 5.54 days, while children with co-detection of HBoV and another respiratory virus were hospitalized for a slightly shorter period, averaging 5.37 days.

Notably, children with high concentrations of HBoV, regardless of whether they had co-infections, experienced longer hospital stays, averaging 5.85 days, whereas those with low concentrations had an average stay of 4.74 days (*p* = 0.04). Spearman’s correlation coefficient of 0.31 suggests a weak positive relationship between viral load and length of stay, meaning that higher HBoV levels were modestly correlated with longer hospitalizations.

For children with mono-detection of HBoV, the *p*-value of 0.06 indicated no statistically significant difference in hospitalization duration based on viral concentration alone. However, the correlation between viral load and length of stay was somewhat stronger in these cases, with a Spearman’s correlation coefficient of 0.35. This result, though not statistically significant, was limited by the relatively small sample size, reducing the statistical power of the analysis.

## 4. Discussion

This study was conducted to explore the clinical characteristics of HBoV infections in children from northwest Croatia over a four-year period. Specifically, we aimed to investigate whether a correlation exists between the severity of clinical presentations and the viral load of HBoV in nasopharyngeal secretions. By analyzing viral concentrations and their association with clinical outcomes, our research sought to provide deeper insights into how HBoV contributes to ARI in children. Our primary goal with this research approach was to enhance the understanding of the pathogenesis of HBoV-related respiratory infections, particularly in pediatric populations, where such infections can lead to a wide spectrum of symptoms—from mild URTI to severe lower respiratory tract complications. By focusing on viral load, we wanted to determine whether high concentrations of HBoV in the respiratory tract were linked to more severe clinical outcomes, such as the need for oxygen supplementation, prolonged hospital stays, and more aggressive treatment measures.

Our study, which included hospitalized children up to 18 years old with both URTI and LRTI, identified an HBoV prevalence of 7.6%. This aligns with global data, which identifies HBoV as a relatively common respiratory virus in children. Guido et al. estimated the global prevalence of HBoV infection at 6% in his work [11], while Polo et al., in their meta-analysis of studies conducted in Europe, reported a slightly higher prevalence of nearly 10% [12]. The latter study also demonstrated that prevalence varies across different studies—a variation likely attributable to differences in study designs, most notably regarding the age range of participants. This highlights the importance of study methodology in accurately determining HBoV prevalence across different populations. [12].

Data on the prevalence of HBoV infections in Croatia come from two key studies. The first, conducted in 2016, focused on children hospitalized with acute respiratory infections (ARIs) and reported a prevalence of 10.3% [20]. The second, a retrospective study from 2018 (and published in 2019), found a significantly higher prevalence of 23.1% [21]. In the present study, prevalence stratification was conducted across age groups, revealing that the prevalence of HBoV strongly depends on the age of the children. The median age of HBoV-positive patients was 16 months, with the youngest child being just seven days old and the oldest 15 years. The majority of HBoV cases were observed in children aged one to three years, accounting for 62% of all cases. Additionally, one-quarter of the children with confirmed HBoV were younger than one year, while only 5% were older than five years. These findings align with previous studies, which also suggest that primary HBoV infections predominantly occur in early childhood [22,23,24]. These consistent findings across different studies reinforce the notion that HBoV primarily affects younger children, suggesting a highly transmissible endemic infection and protection from maternal antibodies.

In our study, 40% of children tested positive for HBoV and presented with URTI, whereas 60% exhibited LRTI. Within the group experiencing upper respiratory tract involvement, the prevailing conditions were febrile respiratory catarrh and acute otitis media. Conversely, among those with lower respiratory tract infections, pneumonia was the primary manifestation, affecting slightly over a third of cases, followed by bronchitis, which impacted one-quarter of the children. This is consistent with other studies conducted on hospitalized children, as noted by Christensen et al. [4]. Al-Iede et al. showed that the three most common symptoms were cough, rhinorrhea, and fever [24]. Additionally, a study by Japanese authors showed that the recovery time for children with acute otitis media is longer in the case of a positive HBoV finding and that HBoV infection may precede secondary bacterial infection of the middle ear caused by *Streptococcus pneumoniae* [25].

During physical examinations, breathing difficulties were observed in one-third of the children who tested positive for HBoV, with dyspnea emerging as the predominant symptom. Tachypnea, coupled with the utilization of accessory respiratory muscles, was noted in 13 cases. Abnormal auscultatory findings were detected in 44% of the children, with wheezing being the most prevalent, consistent with the previous literature indicating HBoV as the third most frequently implicated virus in children with wheezing, trailing behind human rhinovirus and respiratory syncytial virus (RSV) [26]. Recent case reports have also highlighted wheezing as a significant issue in children infected with HBoV, including a report by Lakshimi et al. describing a toddler with HBoV infection exhibiting clear sub-costal retractions and wheezing in both lungs [27].

As already mentioned, our primary motivation for this study was to examine the clinical course of children who tested positive for HBoV and to compare it with the viral load detected in their nasopharyngeal secretions. We utilized quantitative PCR as a diagnostic tool to assess the clinical relevance of HBoV detection in these children, with a focus on identifying acute infections marked by high viral concentrations (i.e., above 10^4^ copies/mL). However, the boundary between high and low virus concentrations is not unequivocally defined. Some authors suggest that viral loads above 10^4^ copies/mL are significant [17], while other studies indicate that, for acute viral infections, it may be more appropriate to set the threshold above 10^6^ copies/mL of viral DNA in nasopharyngeal secretions [28]. It is also important to note that the prolonged post-infectious shedding of HBoV can lead to irrelevant HBoV PCR-positive results, making it challenging to distinguish between mere HBoV detection in children with other conditions and actual HBoV disease. Such considerations underscore the need for further research to better define the relationship between viral load and disease severity in HBoV infections, particularly to guide clinical decision-making in pediatric patients.

Statistical analysis did not uncover a significant variance in viral concentration across all HBoV-positive children (n = 73), irrespective of the site of infection. However, upon narrowing our focus to children with exclusively HBoV detection (n = 13), we observed notably higher viral loads in cases of lower respiratory tract infection. Evaluation of samples where HBoV was the sole pathogen revealed predominantly low concentrations when the infection was localized to the upper respiratory tract. In contrast, in nearly all instances where children developed lower respiratory tract infections, HBoV was detected in high concentrations, surpassing 10^6^ copies per mL. These findings underscore the importance of viral load as a potential marker for the severity of HBoV infections, particularly in lower respiratory tract involvement.

Additionally, an indicator of the severity of clinical presentation in children with detected HBoV, as observed in our study, was the onset of respiratory insufficiency necessitating oxygen supplementation during treatment. Hypoxemia is common in children with lower respiratory tract infections, and SpO_2_ < 94% is present in 73% of children under one year admitted to the hospital for acute bronchiolitis in developed countries [29]. Furthermore, in older children with pneumonia in developing countries, hypoxemia (SpO_2_ < 90%) can be found in 13% of cases. In our study, arterial oxygen saturation was measured in 88% of children with confirmed HBoV, and in 13.7% of children, it was lower than 92%, requiring respiratory support and oxygen supplementation. In nearly all samples from these children (9 out of 10), we observed a high concentration of HBoV, typically surpassing levels of 10^6^ copies/mL. Statistical analysis showed that HBoV concentrations in children requiring oxygen therapy were significantly higher than in children who did not need supplementation (*p* = 0.022). However, the link between HBoV and the onset of hypoxemia is obscured by the frequent co-detection of HBoV with other viruses. In two children experiencing hypoxemia, HBoV was the sole identified pathogen, present in both cases at high concentrations. Among the remaining eight children positive for HBoV who required oxygen supplementation, HBoV was detected alongside other viruses, predominantly rhinovirus. Nevertheless, the presence of HBoV in high concentrations in hypoxemic children, particularly in two instances of singular detection, indicates that this virus holds pathogenic potential, potentially leading to a more severe clinical course of lower respiratory tract infection.

None of the children diagnosed with HBoV in our study developed acute respiratory distress syndrome (ARDS) or required mechanical ventilation. Nonetheless, one child required admission to the intensive care unit (ICU) due to severe pneumonia and prolonged bronchoconstriction. This particular case involved a one-year-old boy who presented with acute respiratory insufficiency caused by significant bronchoconstriction and bilateral pneumonia. Upon admission, the child was immediately treated with frequent inhalation therapy, including salbutamol and ipratropium bromide, supplemented by parenteral corticosteroids and oxygen therapy. Despite these interventions, the child continued to experience partial respiratory insufficiency, with his lowest recorded oxygen saturation level dropping to 75%. He remained in the ICU for four days, receiving high-flow oxygen therapy to stabilize his condition. Multiplex PCR testing exclusively confirmed the presence of HBoV; interestingly, routine antigen testing performed in the ward had initially detected the influenza A virus. This discrepancy between the two tests could be explained by differences in sensitivity and specificity, especially since both nasopharyngeal swab samples were collected within the first five days of symptom onset. Quantitative PCR confirmed the presence of HBoV, with a viral concentration exceeding 10^6^ copies/mL. Hence, we believe that the severe clinical manifestation was primarily attributable to HBoV rather than the influenza virus, which was not validated by a more sensitive and specific molecular technique.

Further supporting the significance of detecting a high concentration of HBoV in this child’s sample is a study by Moesker et al. [30], which found similarly elevated viral loads in seven children who were admitted to the intensive care unit (ICU). In their research, Moesker and colleagues concluded that HBoV mono-infections were responsible for causing severe acute respiratory infections in these cases [30]. Several other reports in the literature document severe cases of respiratory infection caused by HBoV that required intensive care, even in children who were previously healthy with no underlying medical conditions [31,32,33,34,35]. A recent case series involving children aged 1 month to 4 years reported that out of four pediatric cases of HBoV infection, three were linked to acute respiratory failure and spontaneous pneumothorax, while two of these children also exhibited subcutaneous emphysema [35]. Infection with HBoV 1 is also linked to a condition known as plastic bronchitis, a condition marked by the development of casts within the trachea or bronchial tree, leading to reduced lung ventilation [36,37].

Out of 73 children with confirmed HBoV, 13 of them (17.8%) had underlying conditions. When comparing viral concentrations, children with pre-existing conditions were more likely to have higher HBoV viral loads compared to those who were previously healthy (*p* = 0.017 for all HBoV positives and *p* = 0.045 for HBoV mono-infections). This is consistent with the report by Christensen et al., which highlighted several risk factors that may contribute to more severe disease outcomes in children with HBoV: cardiopulmonary diseases, prematurity with chronic lung disease, malignant disease, and immunosuppression [4]. Moreover, a study by Ghietto et al. reported an even higher burden of comorbidities (31%) among HBoV-positive children and concluded that these children are more susceptible to HBoV infection, indicating that virus–host interaction significantly contributes to the development of more severe clinical presentations [38].

Nearly half of the children diagnosed with HBoV in this study were given empirical antibiotic treatment, most commonly broad-spectrum third-generation cephalosporins or a combination of amoxicillin and a beta-lactamase inhibitor. The children who received antimicrobial agents had significantly higher HBoV viral loads compared to those who did not receive such treatments. This observation aligns with the idea that higher HBoV concentrations are associated with more severe clinical symptoms, leading to a greater likelihood of being treated with empiric antibiotic therapy. This is in line with a recent study by García-García et al., which demonstrated that children infected with HBoV, especially those with viral coinfections, were more likely to be treated with antibiotics [39]. Consequently, the availability of rapid and accurate viral diagnostic tools has the potential to significantly decrease the overuse (and misuse) of antibiotics in pediatric patients.

Still, it is important to recognize that previous research has indicated that detecting viruses in children with fever and respiratory symptoms has only a limited effect on reducing antibiotic prescriptions, partly due to difficulties in accurately interpreting positive results. A study from Canada concluded that rapid testing for a wide range of viruses by itself is not particularly effective in managing resource utilization in hospitalized adult patients unless it is combined with approaches to exclude bacterial co-infections [40]. In their findings, only a positive influenza result significantly reduced antibiotic use [40]. Conversely, an observational study by Keske et al. found that antibiotic use was notably reduced in both children and adults following the detection of a viral infection [41]. Our study revealed that higher HBoV concentrations were linked to more frequent antibiotic use; however, additional research is required to assess whether knowledge of HBoV viral loads can aid clinicians in minimizing the unnecessary usage of antimicrobial agents when managing viral respiratory infections.

In our study, children with HBoV infection had shorter hospital stays compared to those with other respiratory viruses. However, children with higher HBoV viral loads remained hospitalized longer than those with lower viral concentrations (*p* = 0.04). When focusing solely on cases of HBoV mono-infections, no significant difference in the length of hospital stay was observed. The Spearman correlation coefficient was low, suggesting a weak positive monotonic relationship between viral load and hospital stay. It is likely that the length of hospitalization is influenced by multiple factors, similar to other acute respiratory infections. For instance, a study on children with RSV demonstrated that predictors of extended hospital stays included younger age, transfers from other facilities, intubation, pre-existing conditions, and the type/location of the hospital [42]. This suggests that other variables beyond viral load play a role in determining the duration of hospitalization for children with HBoV but also other respiratory viral infections.

The limitations of this study include the relatively small number of samples with HBoV as the solely detected pathogen (mono-detection), which was largely due to the frequent co-detection of other respiratory viruses; this, in turn, complicates the statistical analysis, making it difficult to isolate the effects of HBoV alone on clinical outcomes. Furthermore, qPCR yielded negative results for seven samples that were initially positive for HBoV when multiplex PCR was used. It is likely that these samples contained very low levels of HBoV, potentially below the detection threshold of qPCR. Additionally, there is a possibility that the minimal amounts of viral DNA in these samples degraded during storage, even though they were preserved at –80 °C (which is a standard protocol for long-term viral sample preservation). Importantly, in all seven of these samples that tested negative with qPCR, other respiratory viruses were found, which further emphasizes the challenge of accurately attributing clinical symptoms to HBoV when other pathogens are present. Furthermore, while this study focused on viral load and its relationship to clinical outcomes, other important clinical parameters (such as immunological status, nutritional factors, and detailed medical histories) were not fully explored. Lastly, this study’s generalizability may be constrained by its regional focus on children in northwest Croatia. Further studies with larger, more diverse populations across different settings are needed to strengthen the findings.

## 5. Conclusions

In conclusion, our study found that in cases of HBoV mono-infection, children with higher viral loads exhibited more severe clinical presentations. This severity was marked by the progression to lower respiratory tract infections and/or the need for oxygen supplementation. Additionally, children who were treated with empirical antibiotics had significantly higher viral loads, further suggesting that a more severe clinical course was associated with elevated HBoV concentrations.

These findings highlight the importance of considering HBoV as a potential causative agent when evaluating children hospitalized with ARI. The strong correlation between high viral loads in nasopharyngeal secretions and more severe clinical outcomes underscores the value of quantifying viral concentrations in guiding diagnosis and treatment. Measuring HBoV viral load could, thus, be an important diagnostic tool, aiding clinicians in interpreting positive results more accurately and determining the appropriate course of care.

Our study definitely reinforces the need for further investigation into the role of HBoV in respiratory illnesses, especially considering its interaction with other viral pathogens. More comprehensive research is needed to ultimately inform more targeted treatment strategies, reduce unnecessary antibiotic use, and also improve overall patient management in pediatric respiratory infections.

## Figures and Tables

**Table 1:** Primers and probes used for quantitative PCR for HBoV.

Virus	Sequence (5′ → 3′)	Target Gene
HBoV	F: GGA AGA GAC ACT GGC AGA CAA	Non-structural protein 1 (NP-1)
	R: GGG TGT TCC TGA TGA TAT GAG C	
	Probe: FAM-CTG CGG CTC CTG CTC CTG TGA T-BHQ2	

**Table 2:** Clinical presentation of children with detected HBoV (n = 73).

Clinical Diagnosis		N	%
URTI	Febrile respiratory catarrh	12/73	16.4
	Acute otitis media	9/73	12.3
	Pharyngitis	2/73	2.7
	Tonsillitis	4/73	5.5
	Acute Rhinitis	1/73	1.4
	Status febrilis	2/73	4.8
LRTI	Pneumonia	26/73	35.6
	Acute bronchitis	17/73	23.3
	Bronchiolitis	4/73	5.5
Most prominent symptom at admission			
	Breathing difficulties	37/73	50.7
	Elevated temperature	44/73	60.3
	Gastrointestinal (diarrhea, vomiting)	3/73	4.1
Abnormalities found in physical exam			
	Dyspnea	9/73	12.3
	Tachypnea	13/73	17.8
	Hyperemic throat	23/73	31.5
	Abnormal otoscopic finding	9/73	12.3
	Skin changes (urticaria, erythema, purpura, petechiae, generalized rash)	10/73	13.7
Auscultatory findings			
	Normal lung sound	41/73	56.2
	Wheezing	13/73	17.8
	Rales	8/73	11
	Prolonged expiration	6/73	8.2
	Crepitations	5/73	6.8

**Table 3:** Therapeutic measures during hospitalization applied for children positive for HBoV.

		N	%
Antimicrobial therapy		36/73	49.3
	Ceftriaxone	30/73	41.1
	Amoxicillin/clavulanic acid	6/73	8.2
	Azithromycin	3/73	4.1
	Clindamycin	3/73	4.1
	Penicillin	1/73	1.4
Bronchodilators		28/73	38.6
Corticosteroids	Inhaled	7/73	9.6
	Systemic	12/73	16.4
Antihistamines		5/73	6.8
Montelukast		3/73	4.1
Racemic epinephrine		1/73	1.4
Aminophylline		1/73	1.4
Oxygen supplementation		10/73	13.7
Supportive measures (antipyretics, parenteral rehydration, and respiratory physiotherapy)		29/73	39.7

## Data Availability

The data presented in this study are available on reasonable request from the first, last, and/or corresponding author. The data are not publicly available due to the age and privacy of the participants.

## Supplementary Materials

The following supporting information can be downloaded at https://www.mdpi.com/article/10.3390/v16101637/s1, Table S1: Quantitative PCR results for HBoV (n = 73). Figure S1: Total distribution of viral loads in positive HBoV samples (n = 66) (HBoV qPCR: Result of HBoV concentration; Cut-off 1 = 104 copies/mL, Cut-off 2 = 106 copies/mL).
