# Supplementary material for: The Role of Quantitative PCR in Evaluating the Clinical Significance of Human Bocavirus Detection in Children
Source: Viruses. 2024 Oct 19;16(10):1637. doi: 10.3390/v16101637 (PMC11512256; doi:10.3390/v16101637)
Supplement: Supplementary file 1 [file viruses-16-01637-s001.zip › viruses-3262488-supplementary.pdf]

Table S1. Quantitative PCR results for HBoV (n = 73).

Sample	Multiplex PCR Results	Quantitative PCR for HBov (CT Value)	Quantitative PCR for HBoV (Copies/mL)	Infection Localization
1	HBoV	13.06	2.14×10^7	LRTI
2	HBoV, HEV	13.07	1.71×10^7	URTI
3	HBoV	10.27	1.36×10^7	URTI
4	HBoV, HCoV, HRV	10.45	1.24×10^7	URTI
5	HBoV, HRV	10.84	1.01×10^7	URTI
6	HBoV, AdV, HRV	10.87	1.00×10^7	URTI
7	HBoV, RSV B	14.64	9.70×10^6	URTI
8	HBoV	11.3	8.00×10^6	LRTI
9	HBoV, HRV	14.56	7.76×10^6	LRTI
10	HBoV, AdV	15.72	4.81×10^6	LRTI
11	HBoV, HRV	15.51	4.71×10^6	LRTI
12	HBoV	16.05	4.42×10^6	LRTI
13	HBoV, HRV	15.81	3.90×10^6	LRTI
14	HBoV, HRV	16.19	3.30×10^6	LRTI
15	HBoV	17.29	2.30×10^6	LRTI
16	HBoV, AdV	17.3	2.29×10^6	URTI
17	HBoV, AdV	17.42	1.72×10^6	URTI
18	HBoV, HRV	17.87	1.56×10^6	LRTI
19	HBoV, HCoV	17.88	1.55×10^6	LRTI
20	HBoV, HRV	14.7	1.37×10^6	URTI
21	HBoV, HRV	18.23	1.28×10^6	LRTI
22	HBoV	18.5	1.22×10^6	LRTI
23	HBoV, PIV 4, AdV	19.04	8.40×10^5	LRTI
24	HBoV, HCoV, HRV	15.85	7.57×10^5	URTI
25	HBoV, AdV	20.66	3.12×10^5	URTI
26	HBoV, HEV	20.98	3.01×10^5	LRTI
27	HBoV	17.73	2.85×10^5	LRTI
28	HBoV, AdV	21.36	2.17×10^5	URTI
29	HBoV, HRV	21.63	1.88×10^5	LRTI
30	HBoV, RSV A	20.46	1.56×10^5	LRTI
31	HBoV, PIV 3	23.25	8.00×10^4	URTI
32	HBoV, AdV	21.56	7.24×10^4	LRTI
33	HBoV, HRV	23.86	6.63×10^4	LRTI
34	HBoV, HRV	25.03	3.91×10^4	URTI
35	HBoV, AdV, HRV, HCoV OC43, HEV	21.6	3.83×10^4	URTI
36	HBoV, RSV B	24.65	3.82×10^4	LRTI
37	HBoV, HRV	25.39	3.23×10^4	URTI
38	HBoV	26.45	1.48×10^4	LRTI

39	HBoV, HEV	23.58	1.38×10^4	URTI
40	HBoV, AdV, RSV B	24.59	8.16×10^3	URTI
41	HBoV, HRV	28.36	6.77×10^3	URTI
42	HBoV, HRV	35.9	6.29×10^3	URTI
43	HBoV	28.59	4.78×10^3	URTI
44	HBoV, AdV, HRV, HEV,	28.64	4.67×10^3	LRTI
45	HBoV, RSV A	28.95	4.53×10^3	LRTI
46	HBoV	28.7	4.51×10^3	URTI
47	HBoV, HRV, HEV	29.67	2.70×10^3	LRTI
48	HBoV, HCoV OC 43, RSV A	27.75	1.59×10^3	LRTI
49	HBoV, AdV, RSV A	27.77	1.57×10^3	LRTI
50	HBoV, AdV, HRV, RSV B	28.37	1.15×10^3	LRTI
51	HBoV, HRV	28.51	5.86×10^2	LRTI
52	HBoV, RSV A	32.8	5.81×10^2	LRTI
53	HBoV, AdV	32.8	5.81×10^2	URTI
54	HBoV AdV, Flu A	33.29	4.89×10^2	URTI
55	HBoV	30.6	3.61×10^2	URTI
56	HBoV, HRV	33.89	3.55×10^2	LRTI
57	HBoV, PIV 4	34.76	2.03×10^2	URTI
58	HBoV, RSV A, HCoV, HRV	34.92	1.86×10^2	LRTI
59	HBoV	35.29	1.67×10^2	URTI
60	HBoV, RSV A	30.92	1.11×10^2	LRTI
61	HBoV, HMPV	31.7	6.45×10	LRTI
62	HBoV, AdV, HRV, RSV A	31.8	5.99×10	LRTI
63	HBoV, RSV A, HRV	38.06	3.71×10	LRTI
64	HBoV	39	2.22×10	URTI
65	HBoV, RSV B	33.74	1.57×10	LRTI
66	HBoV, HRV, HEV, HCoV	34.43	9.71	LRTI
67	HBoV, HRV	0	0	LRTI
68	HBoV, AdV, HRV	0	0	URTI
69	HBoV, RSV A, HCoV	0	0	LRTI
70	HBoV, PIV 4, AdV, HRV, PIV1	0	0	LRTI
71	HBoV, AdV	0	0	LRTI
72	HBoV, HRV	0	0	LRTI
73	HBoV, HEV, AdV	0	0	URTI

[1] CT, cycle threshold; URTI, upper respiratory tract infection; LRTI, lower respiratory tract infection; AdV, adenovirus; Flu A and B, influenza viruses A and B; HBoV, human bocavirus; HCoV, human coronaviruses; HEV, human enterovirus; HMPV, human metapneumovirus; HRV, human rhinovirus; PiV 1-4, parainfluenza viruses 1–4; RSV A and B, respiratory syncytial virus A and B.

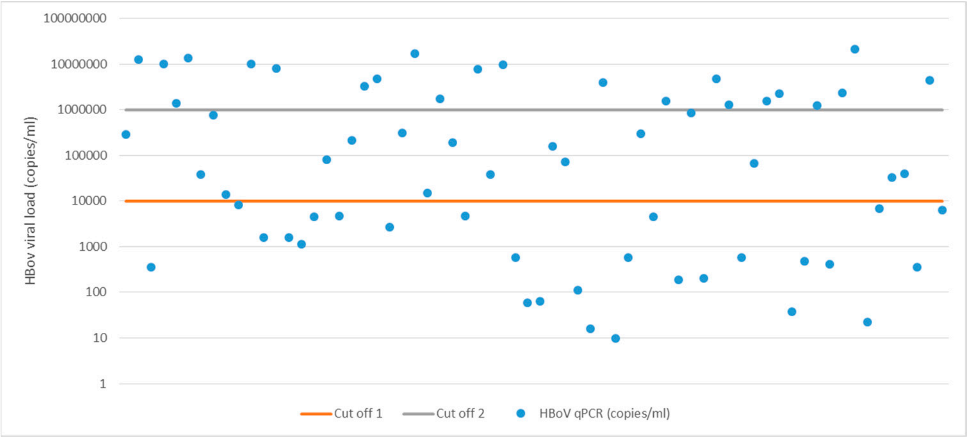

Figure S1. Total distribution of viral loads in positive HBoV samples (n = 66) (HBoV qPCR: Result of HBoV concentration; Cut-off 1 = 10^4 copies/mL, Cut-off 2 = 10^6 copies/mL).
